# Postoperative small intestinal obstruction caused by barbed suture after robot‐assisted laparoscopic sacrocolpopexy

**DOI:** 10.1002/iju5.12677

**Published:** 2023-12-06

**Authors:** Haruka Takagi, Naoki Wada, Shun Morishita, Miyu Ohtani, Takeya Kitta, Hidehiro Kakizaki, Daisuke Kohro, Tatsuya Shonaka

**Affiliations:** ^1^ Department of Renal and Urologic Surgery Asahikawa Medical University Asahikawa Japan; ^2^ Department of Gastrointestinal Surgery Asahikawa Medical University Asahikawa Japan

**Keywords:** barbed suture, robot‐assisted sacrocolpopexy, small intestinal obstruction

## Abstract

**Introduction:**

We present a case of small intestinal obstruction due to a barbed suture used for peritoneal closure during robot‐assisted laparoscopic sacrocolpopexy.

**Case presentation:**

A female patient with pelvic organ prolapse underwent robot‐assisted laparoscopic sacrocolpopexy uneventfully. Intestinal obstruction developed on postoperative Day 4. Conservative treatment with the ileus tube failed to improve abdominal symptoms. The laparoscopic examination on postoperative Day 14 revealed the barbed suture entangled with the small intestinal mesentery. The tail of the barbed suture was laparoscopically detached from the mesentery without damaging the small intestine. The tail of the barbed suture was trimmed; an antiadhesive material was applied to the peritoneal closure line and the trimmed tail of the barbed suture.

**Conclusion:**

We recommend the use of conventional absorbable sutures in the peritoneal cavity because of the potential risk of intestinal obstruction caused by the barbed suture.

**Keynote message**

The use of barbed sutures in the peritoneal cavity may cause intestinal obstruction owing to the entanglement of the barbed suture tail with the intestine or mesentery. Conventional absorbable sutures are recommended in the peritoneal cavity due to the risk of intestinal obstruction caused by barbed sutures.

**Abbreviations & Acronyms**
FDA: Food and Drug Administration
POP: pelvic organ prolapse
RSC: robot‐assisted sacrocolpopexy

## Introduction

Barbed sutures are widely used in general surgery as well as gynecological and urological procedures to reduce the time or prevent the suture from sliding back.^1^ Since the FDA was alerted about mesh complications in 2014, laparoscopic sacrocolpopexy with or without robotic assistance has been replacing transvaginal mesh surgery for POP. Barbed sutures are sometimes used for the peritoneal closure during laparoscopic sacrocolpopexy. Herein, we present a case of postoperative small intestinal obstruction caused by the barbed suture used during RSC.

## Case report

A female patient in her seventies had been suffering from POP for the past 2 years. She visited our department with a complaint of lower urinary tract symptoms, mainly poor urinary stream. Pelvic examination revealed stage IV POP (cystocele) on the POP‐Q system. Her medical history revealed hypertension and diabetes mellitus. She had three vaginal deliveries with no history of abdominal surgery. She was not sexually active. Vaginal pessary treatment was attempted, but it failed to retain and fell out every time. We recommended surgical repair for POP, and she decided to undergo RSC.

**Fig. 1:**
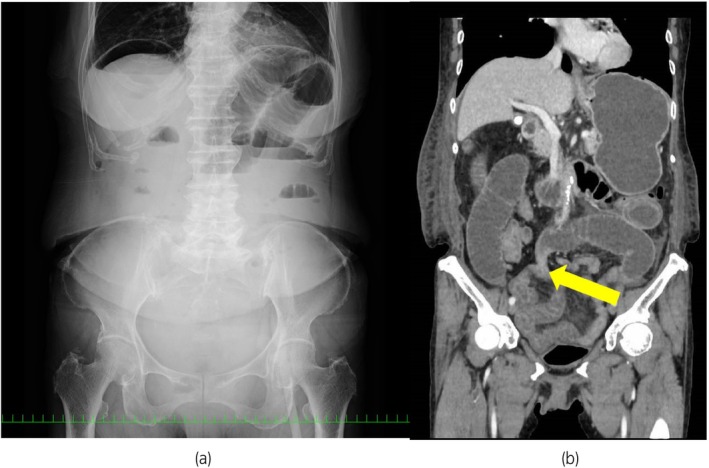
(a) Abdominal X‐ray on the 4th postoperative day showed bowel distension. (b) CT scan on the 11th postoperative day showed distended small bowel and compressed bowel (arrow).

**Fig. 2:**
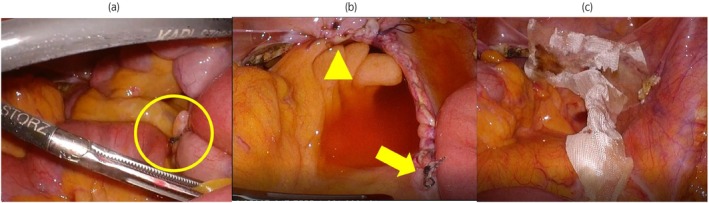
(a) There was adhesion between the tail of V‐Loc™ and the mesentery of the small bowel (circle). (b) There was adhesion between the V‐Loc™ and fatty appendices of sigmoid colon (arrowhead). The arrow showed the tail of the V‐Loc™ entangled to the mesentery. (c) Antiadhesive material was applied to the peritoneal closure line and the trimmed tail of V‐Loc™.

We performed RSC (DaVinci Xi surgical system; Intuitive Surgical, Inc., Sunnyvale, CA, USA) with bilateral salpingo‐oophorectomy and supracervical hysterectomy. We secured the mesh (ORIHIME™; CROWNJUN Co, Chiba, Japan) to the sacrum and closed the peritoneum using a running absorbable 3‐0 V‐Loc™ (Covidien™, Mansfield, MA, USA). The console time was 123 min with minimal bleeding. The patient had vomiting and abdominal distension on postoperative Day 4. She was diagnosed with postoperative intestinal obstruction based on an abdominal X‐ray (Fig. 1a). A computed tomography scan revealed a small intestinal obstruction in the lower right abdomen (Fig. 1b). Conservative treatment with an ileus tube failed to improve the condition. The diagnostic laparoscopy was performed on postoperative Day 14. Laparoscopic examination revealed the entanglement of the tail of V‐Loc™ with the small intestinal mesentery (Fig. 2a). The tail of V‐Loc™ was laparoscopically detached from the mesentery without damaging the small intestine. Furthermore, the V‐Loc™ was adhered to fatty appendices of the sigmoid colon (Fig. 2b). That part was also detached to avoid an internal hernia. The tail of the V‐Loc™ was trimmed, and an antiadhesive material (INTERCEED®; Johnson & Johnson, New Brunswick, NJ, USA) was applied to the peritoneal closure line and the trimmed tail of V‐Loc™ (Fig. 2c). On postoperative Day 1, the ileus tube was removed and the patient was allowed to drink water. She was discharged from the hospital on postoperative Day 3.

## Discussion

Herein, we presented a case of postoperative small intestinal obstruction caused by the barbed suture used during RSC. The barbed suture tail was entangled to the mesentery, causing a small intestinal obstruction. This case report mainly indicates that the use of barbed sutures in the peritoneal cavity may cause intestinal obstruction due to the suture tail entanglement to the intestines or mesentery.

Currently, several barbed sutures are available in the market, including Quill™ knotless tissue closure device (Angiotech™, Vancouver, BC, Canada), V‐Loc™, and Stratafix™ (Ethicon™, Cincinnati, OH, USA). All of them are absorbable monofilament sutures. Data released from each company indicated that V‐Loc™ is made of trimethylene carbonate, whereas both Stratafix™ and Quill™ are made of polydioxanone. The absorption period of V‐Loc™ is approximately 90–100 days and that of Stratafix™ and Quill™ are 182–238 days and 180–210 days, respectively. The number of barbes of V‐Loc™ is approximately 900 per 45 cm, while that of Stratafix™ and Quill™ is unapprised. Talwar *et al*. conducted a randomized comparative study between barbed suture (unspecified) and conventional suture (polyglactin 910) materials for 100 patients who had undergone a laparoscopic total hysterectomy.^2^ Their cohort demonstrated no small intestinal obstruction and a similar incidence of complications between the two groups. They advocated barbed sutures as an excellent alternative to conventional suture materials with the advantages of reduced suturing time and technical difficulty.

Our PubMed search revealed that over 30 cases of small intestinal obstruction owing to barbed sutures have been reported in the English literature (Table 1). V‐Loc™ was used in the majority of the reported cases, followed by Quill™. Initial surgical procedures included inguinal hernia repair, colpopexy, rectopexy, myomectomy, hysterectomy, and Roux‐en‐Y gastric bypass. The median duration from the initial surgeries to the onset of the symptoms caused by small intestinal obstruction was 13 days (1 day–7 months). In most cases, the small intestinal obstruction was resolved by barbed suture detachment or trimming and intestinal release. However, severe strangulated ileus occurred and small intestinal resection was performed in some cases. Yajima *et al*. reported a case of strangulated bowel obstruction caused by V‐Loc™ after robot‐assisted radical cystectomy.^3^ Their case demonstrated a small intestinal strangulation caused by bands formed by fatty appendices of the sigmoid colon and V‐Loc™, which was used to stitch and divide the prostatic venous plexus, causing the internal hernia. Stratafix™ demonstrated no cases of small intestinal obstruction during our PubMed search. Stratafix™ and V‐Loc™ have some structural differences in size and number of barbes. However, the Manufacturer and User Facility Device Experience Database also reported some cases of small intestinal obstruction caused by Stratafix™.^4^ Notably, the use of any kind of barbed suture might be a potential risk for intestinal obstruction.

**Table 1:** Literature lists regarding small bowel obstruction caused by barbed suture threads.

Author	Journal (year; vol.: page)	Initial surgical procedure	Type of barbed suture	Presentation, time from initial surgery	Treatment
Donnellan *et al*.	*J Minim Invasive Gynecol*. 2011; 18: 528	Hysterectomy	Quill™, absorbable	Abdominal pain and vomiting, 30 days	Barbed suture detachment and trimming
Thubert *et al*.	*Int Urogynecol J*. 2011; 22: 761	Sacrocolpopexy	V‐loc™, absorbable	Abdominal pain and symptoms of bowel obstruction, 4 weeks	Midline laparotomy with adhesiolysis and obstruction release
Buchs *et al*.	*Minim Invasive Ther and Allied Technol*. 2012; 21: 369	Promontofixation, inguinal hernia repair, and pelvic floor repair	V‐loc™, absorbable	Diffuse abdominal pain and vomiting, 8 days	Barbed suture trimming and bowel release
Kindinger *et al*.	*Gynecol Surg*. 2012; 9: 357	Myomectomy	V‐loc™, absorbable	Abdominal pain and distension, and loss of appetite, 4 weeks	Release of obstruction
Rombaut *et al*.	*Gynecol Surg*. 2012; 9: 359	Myomectomy	Quill™, unspecified	Abdominal pain and diarrhea, paralytic ileus, 3 weeks	Barbed suture removal and disentanglement
Burchett *et al*.	*J Laparoendosc Adv Surg Tech*. 2013; 23: 632	Myomectomy	V‐loc™, absorbable	Severe abdominal pain and cramping, 4 weeks	Volvulus reduction
Salminen *et al*.	*Tech Coloprotocol*. 2014; 18: 601	Laparoscopic rectopexy	V‐loc™, unspecified	Small bowel obstruction, 1 week	Divided omental band, release of small bowel, and trimming of suture
		Laparoscopic rectopexy	V‐loc™, unspecified	Small bowel obstruction, 1 month	Release of small bowel and trimming of suture
		Laparoscopic sacrocolporectopexy	V‐loc™, unspecified	Small bowel obstruction, 4 months	Release of small bowel and trimming of suture
Filser *et al*.	*Int J Surg Case Rep*. 2015; 8: 193	Bilateral inguinal hernia repair	V‐loc™, absorbable	Abdominal pain, 3 days	Adhesiolysis and removal of suture wire
Köhler *et al*.	*Hernia*. 2015; 19: 389	Laparoscopic inguinal hernia repair	V‐loc™, unspecified	Small bowel obstruction, 13 days	Adhesiolysis and resection of redundant suture
Lee and Wong	*Int J Surg Case Rep*. 2015; 16: 146	Myomectomy	V‐loc™, absorbable	Acute peritonitis, 6 weeks	Adhesiolysis, release of barbed suture from rectum, excision of redundant suture over uterus, and peritoneal washing
Oor *et al*.	*Asian J Endosc Surg*. 2015; 8: 209	Laparoscopic roux‐en‐Y gastric bypass	V‐loc™, absorbable	Abdominal pain and vomiting, 7 days	Removal of free barbed suture end
Segura‐Sampedro *et al*.	*Rev Esp Enferm Dig*. 2015; 107: 677	Rectopexy	V‐loc™, unspecified	Diffuse abdominal pain and distension, 10 days	Strangulated bowel resection and double‐barreled jejunoileosotmy
		Jejunostomy	V‐loc™, absorbable	Abdominal pain, distension, and vomiting, 2 days	Release of adherent suture
Vahanian *et al*.	*Female Pelvic Med Reconstr Surg*. 2015; 21: e11	Hysterectomy	V‐loc™, unspecified	Abdominal pain and projectile vomiting, 22 days	Removal of elongated barbed suture tail and bowel release
		Hysterectomy	V‐loc™, unspecified	Abdominal pain and vomiting, 4 weeks	Removal of elongated barbed suture and bowel release
Chen *et al*.	*Taiwan J Obstet Gynecol*. 2017; 56: 247	Hysterosacropexy	V‐loc™, unspecified	Diffuse abdominal pain and vomiting after meals, 2 days	Release of redundant V‐loc™ suture
Jang *et al*.	*Ann Surg Treat Res*. 2017; 92: 380	Gastrectomy	V‐loc™, absorbable	Abdominal pain and distension, 4 days	Complete closure of hernia and removal of surgical clip
Lee and Yoon	*J Laparoendosc Adv Surg Tech*. 2017; 27: 58	Hepatico‐ jejunostomy	V‐loc™, unspecified	Presentation unknown, 7 months	Hepaticojejunostomy revision
Tagliaferri *et al*.	*J Surg Case Rep*. 2018; 2018: rjy165	Laparoscopic inguinal hernia repair	V‐loc™, unspecified	Diffuse abdominal pain and distension, vomiting after eating, 1 day	Redundant suture trimming and volvulus detorsion
Sartori *et al*.	*G Chir*. 2019; 40: 322	Transabdominal hernia repair	V‐loc™, absorbable	Abdominal pain and vomiting, 3 days	Wire cut and small bowel release
Zipple *et al*.	*Am Surg*. 2020	Laparoscopic inguinal hernia repair	V‐loc™, absorbable	Abdominal pain, vomiting, and mild leukocytosis, 1 day	Removal of elongated barbed suture and bowel release
Man *et al*.	*World J Clin Cases*. 2021; 9: 3696	Laparoscopic inguinal hernia repair	V‐loc™, unspecified	Aggravated abdominal pain, 90 days	Lysis of adhesions and reduction of intestinal volvulus
Zheng *et al*.	*Front Surg*. 2021; 8: 646091	Laparoscopic inguinal hernia repair	V‐loc™, absorbable	Abdominal pain, 3 days	Cutting the barbed suture and volvulus detorsion
Wang *et al*.	*Surg Case Rep*. 2021; 7:161	Laparoscopic inguinal hernia repair	V‐loc™, absorbable	Aggravated abdominal pain, 47 days	Removed the embedded barbed suture in the mesentery
		Laparoscopic inguinal hernia repair	V‐loc™, unspecified	Aggravated abdominal pain, 10 days	The serosal and muscular defect was closed with absorbable sutures
Stabile *et al*.	*Front Surg*. 2021; 8: 626505	Laparoscopic myomectomy	V‐loc™, absorbable	Abdominal pain, nausea, vomiting, and constipation, 7 weeks	Removed elongated barbed suture tail and bowel release
		Laparoscopic sacrocolpopexy	V‐loc™, absorbable	Nausea, vomiting, and constipation, 4 weeks	Barbed suture wire detachment and trimming, bowel release
Limbachiya *et al*.	*CRSLS*. 2022; 9: e2022.00058	Laparoscopic sacrocolpopexy	V‐loc™, unspecified	Abdominal pain, vomiting, and constipation, 5 days	Removed the embedded barbed suture in the mesentery
Yajima *et al*.	*Urol Case Rep*. 2022; 40: 101916	Robot‐assisted radical cystectomy	V‐loc™, unspecified	Abdominal pain and vomiting, 13 days	Releasing the strangulation and resection of small bowel
Qian *et al*.	*Asian J Surg*. 2023; 46: 1815	Inguinal hernia repair	Barbed suture, unspecified	Abdominal pain, 15 days	Cut and removed barbed suture and bowel release
Sarhan *et al*.	*Asian J Endosc Surg*. 2023; 16: 271	Laparoscopic roux‐en‐Y gastric bypass	V‐loc™, unspecified	Abdominal pain, 1 week	Barbed suture detachment and trimming of the tail
Our case	This journal	Robot‐assisted sacrocolpopexy	V‐loc™, absorbable	Vomiting, 4 days	Removed the embedded barbed suture in the mesentery

The use of barbed sutures during minimally invasive surgery is becoming more prominent. The use of barbed suture has a clear advantage of securely reapproximating tissues with less time, cost, and aggravation.^5^ Complications caused by barbed sutures must be avoided despite such benefits. The use of conventional absorbable sutures instead of barbed sutures should be considered, especially in the peritoneal cavity. Cutting the tail of the barbed suture short enough may help prevent entanglement of the suture with other organs if the use of barbed suture is inevitable.^6^, ^7^ Furthermore, applying adhesion barrier materials to prevent direct intestinal contact with the barbed suture is also anoption.^8^

Therefore, we have been using conventional absorbable sutures instead of barbed sutures for peritoneal closure during RSC.

## Conclusions

We should be aware that barbed sutures might cause intestinal obstruction. We recommend the use of conventional absorbable sutures in the peritoneal cavity because of the potential risk of intestinal obstruction caused by barbed sutures.

## Author contributions

Haruka Takagi: Conceptualization; data curation; investigation; writing – original draft. Naoki Wada: Conceptualization; data curation; investigation; writing – original draft. Daisuke Kohro: Data curation; investigation. Tatsuya Shonaka: Data curation; investigation. Miyu Ohtani: Data curation; investigation. Shun Morishita: Data curation; investigation. Takeya Kitta: Data curation; investigation. Hidehiro Kakizaki: Conceptualization; supervision; writing – review and editing.

## Conflict of interest

The authors declare no conflict of interest.

## Approval of the research protocol by an Institutional Reviewer Board

Not applicable.

## Informed consent

Written informed consent was obtained from the patient.

## Registry and the Registration No. of the study/trial

Not applicable.
